# Climate change and liver disease: a mini review

**DOI:** 10.3389/fgstr.2024.1334877

**Published:** 2024-05-15

**Authors:** Tiago Ribeiro, Guilherme Macedo

**Affiliations:** ^1^ Department of Gastroenterology, Centro Hospitalar Universitário de São João, Porto, Portugal; ^2^ World Gastroenterology Organisation (WGO) Gastroenterology and Hepatology Training Center, Porto, Portugal; ^3^ Faculty of Medicine of the University of Porto, Porto, Portugal

**Keywords:** climate change, hepatology, medical waste, metabolic dysfunction-associated steatotic liver disease, alcohol-related liver disease

## Abstract

Climate change poses a growing threat to human health and well-being, with emerging evidence pointing to its intricate relationship with liver diseases. Indeed, climate change influences liver diseases through various direct and indirect mechanisms. Alcohol-related liver disease, Metabolic dysfunction-associated liver disease (MASLD), and viral hepatitis are the three most common causes of liver disease, and all are susceptible to the effects of climate change. Shifts in dietary habits driven by altered food availability, substance abuse exacerbated by social instability, extreme weather events affecting healthcare access, and the emergence of waterborne infections are among the factors exacerbating liver disease incidence and severity. On the flip side, healthcare systems, including liver units, significantly contribute to climate change through energy consumption, medical waste, and transportation emissions. The need for sustainable healthcare practices, telemedicine, and waste reduction strategies is becoming increasingly evident. Recognizing this intricate interplay and addressing the dual interaction between climate change and liver disease is imperative for safeguarding human health and reducing the environmental footprint of healthcare facilities. As climate change continues to unfold, understanding its implications for liver disease is critical for public health and environmental sustainability.

## 1 Introduction

The climate crisis resulting from human activity is one of the main challenges of our time. Indeed, rising temperatures have a significant impact on several sectors of society, and the potential effects on the health of populations are, conceivably, one of the main concerns. In fact, climate change has been identified as the biggest threat to global health in the 21^st^ century. Besides directly affecting the health condition of each individual, climate change impacts the social and environmental determinants of health (1–3).

The healthcare sector is a key player for fostering good health outcomes for the populations. Nevertheless, when considering its environmental impact, we are faced with the paradox regarding the fact the healthcare sector constitutes one of the largest greenhouse gases (GHG) emitters. This reality is easily understandable when we consider that western healthcare systems are large employers and may account for almost 10% of national emissions (4, 5).

Liver disease accounts for 4% of all annual deaths worldwide (6). The incidence of liver disease is intrinsically dependent on human behavior, social context as well as environmental exposures. While these well-defined risk factors open the door for the institution of preventive measures, climate change has the potential to influence all determinants of liver disease, thus contributing to the anticipated increase in the global burden of liver disease (6).

While the available evidence remains scarce and at the level of conjectures, the most relevant scientific societies are increasingly becoming aware of the importance of this situation (7). This short review aims to provide an outlook of the potential impact of climate change on liver disease, as well as on the care for the patient with liver disease and the organization of hepatology centers. Most importantly, this work aims to bring awareness of this increasingly relevant challenge to the medical community, and particularly for hepatologists.

## 2 Dual interaction between climate change and healthcare

The current climate crises impose an additional stress factor on already saturated healthcare care systems. Indeed, the increase in global temperature is expected to disrupt the normal functioning of healthcare delivery by increasing the burden of disease, disrupting healthcare facilities and supply chains, and increasing the susceptibility for more severe forms of disease (8). Nonetheless, the interaction between healthcare and climate change is bidirectional, with healthcare both contributing to and being impacted by climate change.

### 2.1 Healthcare contribution to climate change

#### 2.1.1 Energy consumption

Modern healthcare facilities, including hospitals and clinics, are energy-intensive environments. In fact, large energy requirements are imposed by the need to ensure the functioning of medical equipment, as well as provide ventilation, heating and cooling of facilities. In developed countries, the amount of energy consumption has increased in parallel with an increase in healthcare budget in percentage of GDP (9). This energy consumption leads to greenhouse gas emissions, primarily carbon dioxide (CO_2_). The number of healthcare facilities has been increasing in number and size in the Western hemisphere, and the intensity of energy consumption is calculated to surpass that of commercial buildings three-fold (10). In the US, around 60% of all energy expenditure in healthcare facilities is related to heating and cooling and natural gas is the main source of energy for 90% of buildings (10). Moreover, despite a decrease in energy consumption over the last years for commercial buildings, this reduction was not observed in hospitals, thus underscoring the need for the development of more efficient energy expenditure.

Besides the environmental impact of energy expenditure of healthcare facilities, an increasing attention has been given to the full life cycle of activities related to healthcare delivery. Indeed, a recent study calculated that 45% of GHG emissions of an endoscopy center were related to patient and staff travels, followed by the production and disposal of medical equipment (32%) (11).

#### 2.1.2 Pharmaceuticals and medical waste

The production, use, and disposal of pharmaceuticals contribute to environmental pollution. Additionally, medical waste generated by healthcare facilities, including hazardous materials, requires proper disposal to minimize environmental contamination. Developed countries show higher rates of waste generation, in relation with higher spends in terms of GDP in healthcare (9). Healthcare waste (HCW) accounts for 1-2% of total urban waste (12), and 15% of all HCW is considered hazardous. Practices regarding healthcare waste management are extremely heterogeneous over the globe. Inadequate human resource training raises a significant problem in the treatment of HCW, particularly in developing countries, which poses risks to public health (13).

### 2.2 Healthcare’s vulnerability to climate change

#### 2.2.1 Disruption of healthcare structure

The increased frequency of extreme weather events can cause damage to healthcare facilities, thus limiting patients’ access and quality of care (8). Besides, these events may compromise communication routes, thus disrupting supply chains. Additionally, the impact of anthropogenic climate change is expected to generate economic struggling, thus limiting the investment in the healthcare system. Importantly, the increase in global temperatures and the rise of sea levels may lead to massive population migration movements, therefore increasing the burden on health systems. Moreover, social instability caused by resource scarcity may lead to conflict with risk of destruction healthcare facilities (14).

#### 2.2.2 Vector-borne diseases

The change in climate patterns can modify the geographical distribution of disease vectors, thus bringing diseases from tropical areas to previously temperate climates (e.g. Dengue fever, Malaria). Moreover, economic and food insecurity, as well as mass migratory movements will lead to the establishment of new human settlements (e.g. refugee camps), potentially leading to overcrowding and increased exposure to contaminated water and disease-spreading vectors (14).

#### 2.2.3 Heat-related illnesses

Rising temperatures can lead to more frequent and severe heatwaves, causing heat-related illnesses and, importantly, decompensating preexisting comorbid conditions, including respiratory, cardiovascular and hepatic disease (15–17). The expected increase in admissions due to heat-related illnesses will increase the burden on healthcare systems, which is particularly worrisome for hotter areas, frequently with low-resource healthcare systems.

### 2.3 The impact of climate change on liver disease

The interaction between the climate crisis and the care of patients with liver disease is complex, and potentially bidirectional. A direct impact of the climate in liver health has been shown as a component of heat-related illnesses. Indeed, heat stroke can result in hepatocellular injury due to liver hypoperfusion, leading to acute liver injury and acute liver failure (18). Nevertheless, the indirect effects of climate on liver disease, by inducing modifications on patients’ environments, thereby modifying risk factors and the natural evolution of common liver disease etiologies. On the other direction, patients with liver disease often requires multiple visits, follow-up tests, resource-consuming and chronic medication.

#### 2.3.1 Alcohol-related liver disease

Alcohol use is the leading cause of liver disease in the world, and accounts for 60% all cases of cirrhosis in the western hemisphere (6). The prevalence of alcohol use disorder varies greatly between regions but appears higher in high-income countries (6). Several factors influence pathologic patterns of alcohol consumption. The relation between climate change and mental health has been previously acknowledged (19). Climate change generates several stressors, including depression from destruction resulting from extreme weather events, economic difficulties, food insecurity, and displacement. All these factors can contribute to impair the mental health of individuals. In reaction, people may turn to alcohol as a coping mechanism, increasing the risk of heavy drinking and the development of alcoholic liver disease. Furthermore, there is evidence correlating an increase in atmospheric temperature and more deleterious patterns of alcohol use. Indeed, increasing temperatures have been associated with an increase in overall alcohol use, as well as acute poisoning by alcohol and emergency department visits due to alcohol use disorder (20, 21). Inversely, alcohol consumption contributes with a large volume of GHG emissions, therefore contributing to the anthropogenic climate change. Indeed, a Swedish study estimated that the alcohol industry accounted for 3% of GHG emissions, producing an equivalent of 2.4 kg of CO_2_ per litter (22).

Finally, with increasing frequency of extreme weather events, such as heat waves, the incidence of heat-related illnesses is expected to increase (23). Alcohol may interfere with the perception of heat and regulatory thermoregulation mechanisms, thus increasing the propensity for dehydration, making individuals more susceptible to heat-related complications during extreme weather events, which may further exacerbate the negative effects of alcohol on the liver (24, 25).

#### 2.3.2 Metabolic dysfunction-associated steatotic liver disease

The designation of Metabolic dysfunction-associated steatotic liver disease (MASLD) replaced the term “non-alcoholic fatty liver disease” (26). This etiology is associated with metabolic risk factors, namely the presence of central obesity and diabetes mellitus, affecting 32% of the of the world’s population, and corresponding to the second largest indication for liver transplantation (6).

The increase in the prevalence of MASLD parallels that of obesity and metabolic syndrome (27). The dietary patterns of population are expected to be severely impacted by the anthropogenic climate change (28). These modifications, simultaneously with current patterns of food production, will contribute to the destruction of habitats and loss of biodiversity, which will ultimately limit food availability (29). Food and economic insecurity promote the consumption of highly processed foodstuff, frequently inexpensive, and which contribute greatly to worse metabolic outcomes, exacerbating the obesity epidemics, a major risk factor for MASLD. Moreover, extreme weather events can disrupt food supply chains, leading to food shortages and further influencing dietary habits. Air pollution, particularly in highly populated areas with high concentrations of fine particulate matter may increase the risk of MASLD, as it promotes oxidative stress, systemic inflammation, insulin resistance and impair glucose and triglyceride metabolism (30, 31).

The increase in global temperature, as well as the increasingly prevalent heat waves, are likely to foment sedentarism, as outdoor physical activity becomes unpleasant or contraindicated. Moreover, this may further contribute to feed a vicious circle, whereby a lower predisposition for physical activity will probably contribute an increase in the use of motorized transportation, thus increasing GHG emissions.

#### 2.3.3 Viral hepatitis

Global warming may contribute to increase the frequency of viral hepatitis, both those related to waterborne infections (hepatitis A and E) or those transmitted by parenteral or sexual contact (hepatitis B and C). Indeed, the increasing prevalence of extreme weather events, causing more frequent flooding events may exacerbate the spread of both hepatitis A and E. Moreover, mass population movements from people living in more endangered areas, generating large settlements with poor sanitation, also predisposes to the spread of hepatitis A and E. Besides the potential for increasing number of infections, rising temperatures have been shown to increase in viral pathogenicity, inducing viral replication and mutations (32). The potential for increase in transmission of hepatitis B and C will more probably be a result of the social and economic instability predicted to happen due to the climate crisis. Indeed, social and economic hardship, combined with the dismantlement of healthcare or social facilities for a close contact with vulnerable patients, may increase the exposure to risk factors for hepatitis B and C, namely the use of intravenous drugs.

#### 2.3.4 Other causes of liver disease

The increase in global temperatures has been shown to modulate the typical seasonal pattern of parasitic infections. Fasciolosis is the standard of parasitical liver infection. There is evidence showing climate change driven modifications to the seasonal dynamic of *Fasciola hepatica* infection (33). Moreover, high-risk areas of infection are predicted to extend to previously low-risk areas (33).

Aflatoxin B1, produced by mycotoxigenic fungi species, including *Aspergillus flavus* and *Aspergillus parasiticus* is a known co-factor for hepatocellular carcinoma (34). These molds are particularly prevalent in subtropical regions. Climate change, with increasing ambient temperatures, is driving an increase in the area of prevalence of mycotoxigenic moulds, which are becoming an increasing concern in Europe and North America (35).

## 3 Call for action for sustainable hepatology

The acknowledgement that medical practice has a significant impact in climate change, scientific societies have been issuing position statements, engaging patients, and healthcare staff to promote more sustainable habits and clinical practices. For Gastroenterology and Hepatology, this movement was initiated by developing standards for the adoption of a “greener” endoscopy practice. More recently, a joint group by the British Association for the Study of the Liver (BASL), the European Association for the Study of the Liver (EASL) and the American Association for the Study of Liver Diseases (AASLD) has issued a commentary linking climate change with the care for patients with liver disease (7). This joint position statement offers some guidance regarding the practice of sustainable hepatology. The authors focused on four pillars for sustainable hepatology practice, which included: prevention of disease, patient empowerment, lean service delivery and low carbon alternatives (**Table 1**).

**Table 1:** Actions from sustainable hepatology.

Target audience	Prevention	Patient empowerment	Lean service delivery	Low carbon alternatives
Medical staff	Patient and colleague education Advise on merits of plant-based diet Advise on benefits of physical activity Encourage risk reduction measures for viral hepatitis Social prescribing Engage at patients’ communities	Encourage use of apps to monitor health Advance care planning Involve patients in the development of accessible and sustainable hepatology services	Selecting suitable candidates for hepatocellular carcinoma surveillance De-prescribing Favor noninvasive testing Advance care planning Social prescribing	Virtual consultations Request electronic journals rather than in print Attend some meetings virtually Use of Baveno criteria to determine need for variceal screening Transnasal office-based endoscopy for variceal screening
Departments	Patient and colleague education	Adopt patient led follow-up when possible	Establish abnormal LFT protocols Reduce low-value contacts in outpatients – encourage telemedicine when possible Support green endoscopy	Virtual consultations where appropriate
Scientific societies	Advocate for minimum unit pricing Advocate for ban on marketing of unhealthy foods Advocate for easy access to active transport and exercise for all Advocate for reduction in inequality in access to health Educate members on link between climate and health	Develop technology to monitor disease decompensation Involve patient organizations in the development of sustainable hepatology services	Publish guidance Promote examples of good practice Establish an evidence base for lean service delivery	Deliver more digital education Deliver sustainable conferences Undertake research into low-carbon alternatives specific to hepatology
Industry	Education			Reduce hardcopy material in advertisements Reduce waste in packaging of medications and devices Provide carbon footprint information regarding manufacturing processes Reduce staff travel, including in-person visits to medical centers

***Adapted from Donnelly et al.*** (7).

The incidence of liver disease is intricately dependent of behavioral risk factors, which are often hazardous to the planet themselves. For example, changing the focus of western diet from animal protein and highly processed foods to a plant-based diet would contribute both to decrease the carbon footprint of human feeding but also help mitigating risk factors for liver disease, particularly for MASLD. Additionally, fomenting the practice of physical activity and discouraging the use of motorized transportation would simultaneously contributing to reduce the incidence of MASLD and GHG emissions.

Modern clinical practice is increasingly adopting non-invasive or minimally invasive for the diagnosis and monitoring of diseases. These alternatives frequently have a lower environmental impact comparing to the standard of care. For example, implementing telemedicine appointments for chronically compensated cirrhotic patients, combined with non-invasive tests, may enhance patient compliance and comfort, while reducing the frequency of visits to the clinic. A major example of the utility of non-invasive screening tests in hepatology is the identification of patients with clinically significant portal hypertension using the Baveno VII criteria (36). Selecting patients for variceal screening will help reduce the need for esophagogastroduodenoscopies, therefore mitigating the volume of waste related to endoscopic procedures. The development of standardized and coordinated strategy within each healthcare system will ultimately contribute to improve results. Additionally, besides the reorganization of the healthcare system, the multidirectional interaction between climate change and liver health demands a coordinated and multidisciplinary approach, taking into consideration the insights from scientific societies, patient associations, public health authorities, policy makers, as well as the industry, both medical and alimentary.

The complex interplay between climate change and liver diseases underscores the far-reaching implications of environmental factors on human health. Climate change impacts the incidence, severity, and patient care of liver diseases, including alcohol-related liver disease, MASLD and viral hepatitis. Additionally, liver disease care itself contributes to climate change through energy consumption, pharmaceutical and medical waste, and transportation emissions. Addressing this dual interaction requires a multifaceted approach. Sustainable healthcare practices, telemedicine, waste reduction and recycling, public health strategies, and patient education can collectively reduce the environmental impact of liver disease care while improving patient outcomes. Recognizing the intricate relationship between climate change and liver diseases is essential for promoting a healthier and more sustainable future.

## Author contributions

TR: Conceptualization, Investigation, Writing – original draft, Writing – review & editing. GM: Conceptualization, Supervision, Writing – review & editing.
